# Salicine

**Published:** 1881-06

**Authors:** 


Salicine.—We are glad to see the profession awaking to the import-auce of this valuable remedy. After its failure to reach the expectations of those who, some years ago, calculated largely upon it as a substitute of quinine in the management of malarial fever, it fell into disuse to a large extent, and it is only since the advent of salicylic acid, and its wonderful achievements in rheumatism, that many members of the profession have been disposed to call it forth from its long repose, and to press it into active service in rheumatism and kindred affections. We have used it for many years as a remedy in certain forms of neuralgia, angina pectoris, etc., and as an *anti-pyretic*, in the hot stage of fever. In the pyrexia of fever, we have long regarded it as without a peer, so far as our present knowledge of the action of drugs extends in that direction; and the experience of the past few years, has convinced us that when associated with certain adjuvants, as indicated by the stage, symptoms, etc., of rheumatism, that it is probably the most valuable remedy we possess in the management of that hydra-headed monster.

